# Synthesis and photophysical properties of benzoxazolyl-imidazole and benzothiazolyl-imidazole conjugates†

**DOI:** 10.1039/d1ra08342b

**Published:** 2021-12-17

**Authors:** Hsing-Yu Chen, Chen-Chen Yao, Tzu-Yu Tseng, Yao-Chun Yeh, He-Shin Huang, Mei-Yu Yeh

**Affiliations:** Department of Chemistry, Chung Yuan Christian University No. 200, Zhongbei Rd., Zhongli Dist. Taoyuan City 320314 Taiwan myyeh@cycu.edu.tw

## Abstract

Materials that have higher fluorescence emission in the solid state than molecules in solution have recently been paid more attention by the scientific community due to their potential applications in various fields. In this work, we newly synthesized benzoxazolyl-imidazole and benzothiazolyl-imidazole conjugates, which show aggregation-induced emission (AIE) features in their solid and aggregate states. It was found that oxygen and sulfur substitutions can dramatically influence the molecular structures and polarities of the dyes, leading to different degrees of the AIE phenomenon. The benzothiazolyl-imidazole molecule has lower polarity compared to that of benzoxazolyl-imidazole; therefore, the dye bearing a benzothiazolyl group shows higher emission intensity and dual emission in aqueous solution. Theoretical calculation results suggest that the benzothiazolyl-imidazole molecules might have electrostatic interactions between sulfur and nitrogen atoms, explaining the experimental observations of lower critical aggregation concentration and photophysical properties both in solution and in the solid state. The theoretical calculations agree with the experimental data, thus demonstrating a potent strategy to gain a deep understanding of the structure–property relationships to design solid-state fluorescent materials.

## Abstract 2

The effect of heteroatoms on the structural and photophysical properties of donor-π-acceptor molecules, comprising imidazole and benzoxazolyl as well as imidazole and benzothiazolyl units, was investigated.

## Graphical abstract

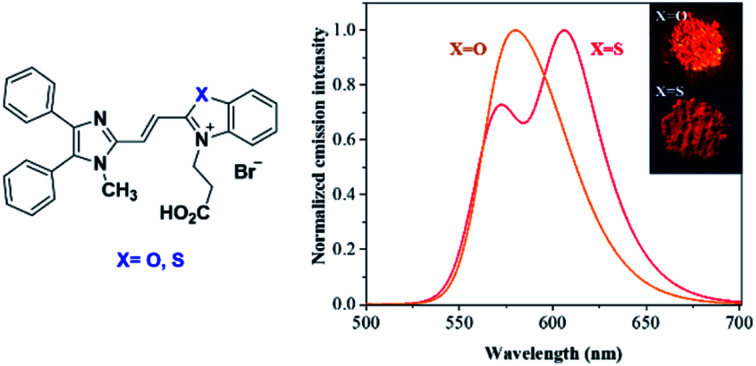

## 1. Introduction

Materials that have higher fluorescence emission in the solid and aggregate state than that of molecules in solution have recently been paid more attention by the scientific community due to their potential applications in various fields, including explosive detection, optoelectronics, photonics, sensing, biomedical applications and so on.^1–11^ The concept of aggregation-induced emission (AIE) was discovered in 2001 and since then numerous AIEgens have been developed by researchers, academics and others.^12^ Until now, several interesting types of AIEgen based on tetraphenylethene,^13–15^ hexaphenylsilole,^16,17^ tetraphenylpyrazine,^18,19^ distyrylanthracene,^20,21^ boron diiminates^22^ and imidazole^23^ have been explored.

Among these chromophores, imidazole-based molecules were relatively rarely investigated as AIEgens, instead they were extensively utilized for anticancer agents, antibacterial agents, catalysts, dyes and ionic liquids.^24–31^ On the other hand, cyanines are a popular class of π-conjugated donor–acceptor (D–A) chromophores with a characteristic of charged structures. The scientific interest in cyanine dyes is mainly related to their excellent spectral properties, for instance good fluorescence quantum yields, high molar extinction coefficients, broad wavelength tunabilities, stability, and increased sensitivity.^32,33^ Besides, benzoxazolyl and benzothiazolyl derivatives, such as oxazole yellow and thiazole orange, have been proven to achieve selective labeling of cancer cells due to their high affinity.^34–36^ However, they have weak fluorescence in their aggregated state, limiting their applications.^37–39^ Thus, this work aims to develop new types of donor–acceptor-conjugated cyanine-like molecules, which show AIE features in their solid and aggregate states. Herein, the newly discovered D-π-A molecules, comprised of imidazole and benzoxazolyl as well as imidazole and benzothiazolyl units were designed and synthesized. The effect of heteroatoms (oxygen and sulfur) on the photophysical properties and molecular geometries were investigated. It was found that the oxygen and sulfur substitutions can dramatically influence molecular structures and polarities of the dyes, leading to the different degree of AIE phenomenon. Since the benzothiazolyl-imidazole molecule has lower polarity than benzoxazolyl-imidazole, the dye bearing benzothiazolyl group shows higher emission intensity and dual emission in aqueous solution, which accompanying the more minor AIE enhancement and red solid is observed. Furthermore, the theoretical calculations agree with the experimental observations, thus demonstrating a potent strategy to gain a deep understanding of the structure–property relationship to design solid-state fluorescent materials.

## 2. Methods and materials

### 2.1. Synthesis of dyes

#### 2.1.1. Synthesis of 6a

Compound 3 (ref. 23) (0.14 mmol, 36.7 mg) and 5a^40^ (0.14 mmol, 40.1 mg) were dissolved in dry ethanol with trace amount of pyridine, and the reaction mixture was heated to reflux under nitrogen for 12 hours. After cooled to room temperature, the reaction mixture was concentrated by rotary evaporator and the residue was precipitated with hexane, then washed with acetonitrile to obtain compound 6a (23.7 mg, 32%) as an orange-red solid. ^1^H NMR (400 MHz, DMSO-*d*_6_): *δ* = 3.02 (t, 2H, CH_2_, *J* = 6.8 Hz), 3.74 (s, 3H, CH), 5.11 (t, 2H, CH_2_, *J* = 6.8 Hz), 7.23–7.27 (m, 3H, 3CH), 7.45–7.79 (m, 4H, 4CH), 7.58–7.61 (m, 3H, 3CH), 7.81 (t, 1H, CH, *J* = 8.2 Hz), 7.90 (t, 1H, CH, *J* = 8.2 Hz), 8.12 (d, 1H, CH, *J* = 15.2 Hz), 8.18 (d, 1H, CH, *J* = 15.2 Hz), 8.35 (d, 1H, CH, *J* = 8.2 Hz), 8.47 (d, 1H, CH, *J* = 8.2 Hz). ^13^C NMR (100 MHz, DMSO-*d*_6_): *δ* = 32.12, 33.06, 45.40, 113.89, 117.38, 124.85, 127.23, 127.87, 128.57, 128.78, 128.76, 130.05, 130.98, 133.26, 133.90, 135.11, 141.43, 141.65, 142.85, 171.82, 171.96. HRMS (ESI^−^) *m*/*z* for C_28_H_23_BrN_3_O_3_, calcd 528.09228, found 528.27682.

#### 2.1.2. Synthesis of 6b

In a manner similar to that described above, a solution of 3 (ref. 23) (0.14 mmol, 36.7 mg), 5b^41^ (0.14 mmol, 42.3 mg) and the catalytic amount of pyridine in dry ethanol to yield compound 6b (19.0 mg, 25%) as red solid. ^1^H NMR (400 MHz, DMSO-*d*_6_): *δ* = 3.00 (t, 2H, CH_2_, *J* = 6.3 Hz), 3.06 (s, 3H, CH), 5.08 (t, 2H, CH_2_, *J* = 6.3 Hz), 7.21–7.29 (m, 3H, 3CH), 7.43–7.46 (m, 4H, 4CH), 7.56–7.57 (m, 3H, 3CH), 7.79 (t, 1H, CH, *J* = 7.5 Hz), 7.87 (t, 1H, CH, *J* = 7.5 Hz), 8.10 (d, 1H, CH, *J* = 14.8 Hz), 8.15 (d, 1H, CH, *J* = 14.8 Hz), 8.31 (d, 1H, CH, *J* = 8.1 Hz), 8.43 (d, 1H, CH, *J* = 8.1 Hz). ^13^C NMR (100 MHz, DMSO-*d*_6_): *δ* = 32.11, 33.04, 45.38, 113.86, 117.38, 124.85, 127.21, 127.85, 128.56, 128.77, 128.85, 129.70, 129.75, 130.04, 130.97, 133.29, 133.93, 135.10, 141.43, 141.65, 142.85, 171.83, 171.98. HRMS (ESI^−^) *m*/*z* for C_28_H_23_BrN_3_O_2_S_1_, calcd 544.06943, found 544.07623.

### 2.2. Characterizations

The ^1^H and ^13^C NMR spectra were conducted with Bruker Avance NEO 400 MHz NMR spectrometer using DMSO-*d*_6_ as the solvent. The UV-vis absorption and fluorescence emission spectra were recorded on Shimadzu UV-2550 spectrometer and Horiba FluoroMax®-4 spectrometer, respectively. Emission spectra of 6a and 6b were observed for the excitation wavelengths of 470 nm and 475 nm, respectively. Micro fluorometer cuvettes (1 mm light path, 0.35 mL volume) were used to avoid reabsorption of emission from the sample.

### 2.3. Computational methods

All the calculations were performed with the Gaussian98 program package.^42^ The geometric structures of molecules 6a and 6b were optimized with density functional theory (DFT) using the B3LYP functional (Becke's three-parameter hybrid functional using the correlation functional of Lee, Yang, and Parr, which includes both local and non-local terms correlation functionals) and the 6-31G(d,p) basis set.^43^ The Hartree–Fock (HF)/6-31G(d,p) methods were also carried out to compare the DFT results and the experimental measurements. The transition energies were calculated at the TD-DFT/B3LYP level of approximation by using the ground state DFT/B3LYP/6-31G(d,p) and HF/6-31G(d,p) geometries, respectively.

## 3. Results and discussion

### 3.1. Synthesis

**Scheme 1:**
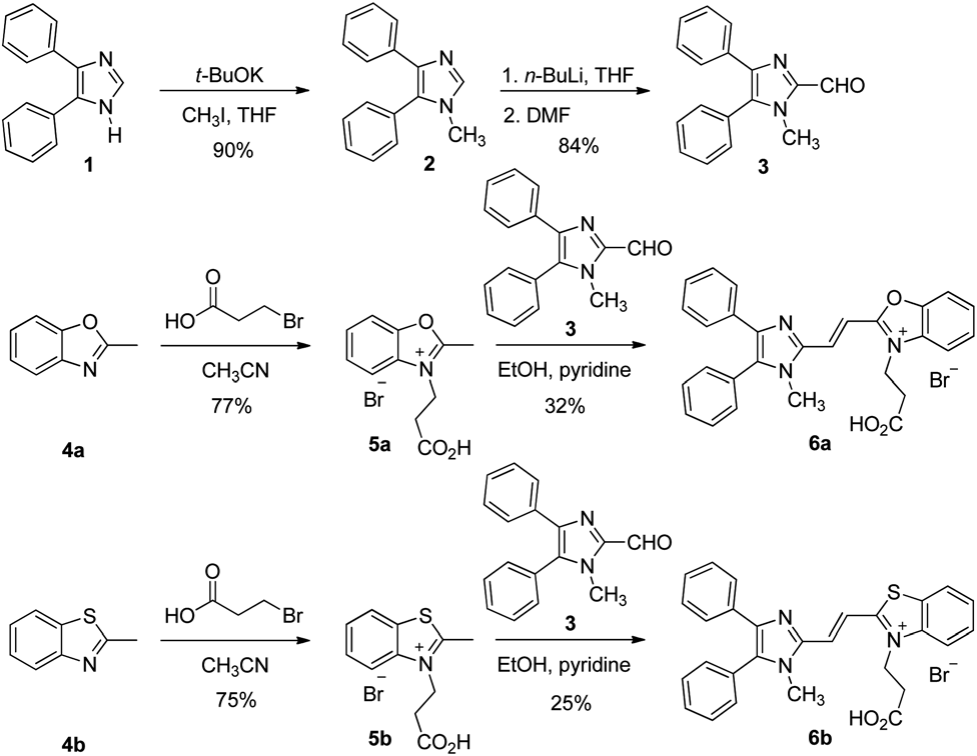
Synthetic routes of dyes 6a and 6b.

The synthetic routes of dyes 6a and 6b are displayed in Scheme 1. Compounds 3, 5a and 5b were synthesized according to literature procedures.^23,40,41^ 1-Methyl-4,5-diphenylimidazole (2) was produced by methylation of 4,5-diphenylimidazole (1). Subsequently, compound 3 was furnished by formylation of 2 with dimethylformamide (DMF).^23^ Meanwhile, the heterocyclic quaternary salts of 5a and 5b were carried out in the reaction of the 3-bromopropionic acid with 2-methylbenzoxazole (4a) and 2-methylbenzothiazole (4b), respectively.^40,41^ The newly discovered dyes of 6a and 6b were prepared through condensation reactions of 3 and 4a as well as 3 and 4b in the presence of pyridine, respectively. The identification of the new compounds was determined by ^1^H NMR, ^13^C NMR and high resolution mass spectrometry (see Methods and materials section for a full description and data shown in the ESI†).

### 3.2. Spectral properties

**Fig. 1:**
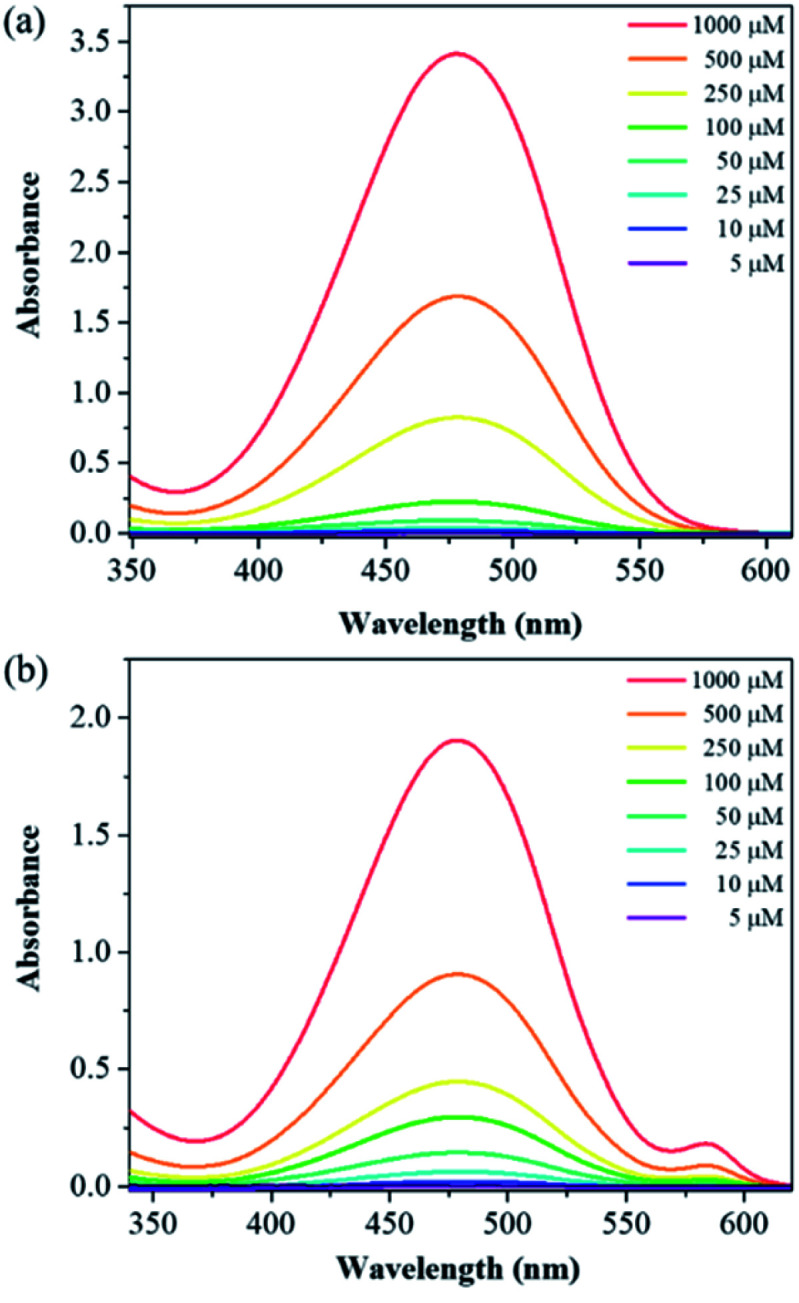
Concentration-dependent UV-vis absorption spectra of (a) 6a and (b) 6b in DMSO.

**Fig. 2:**
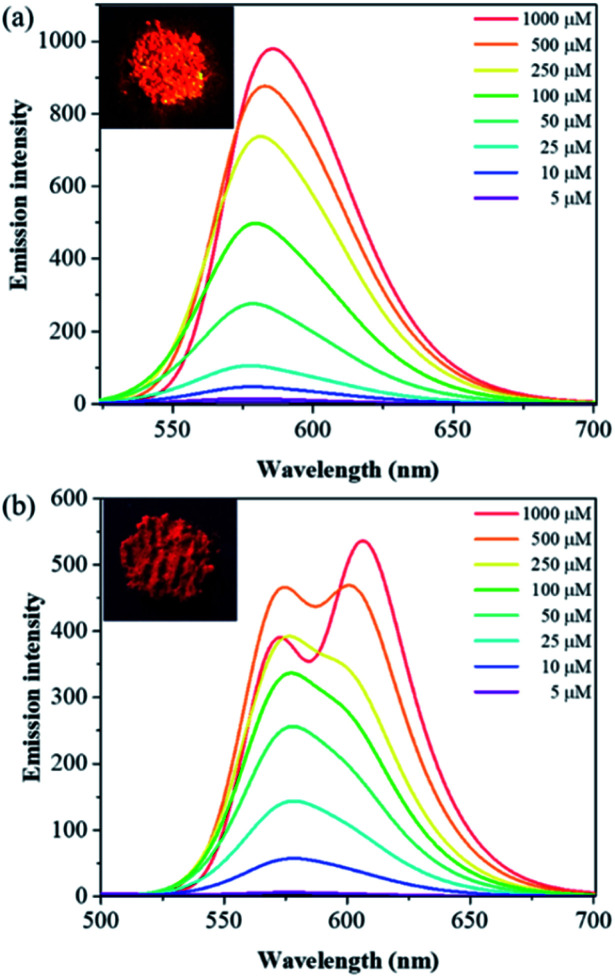
Concentration-dependent fluorescence emission spectra of (a) 6a and (b) 6b in DMSO with excitation at wavelengths of 470 and 475 nm, respectively. The insets are the photographs of solid powders of 6a and 6b taken under UV illumination.

With compounds 6a and 6b in hand, we investigated the effect of atom substitutions (oxygen and sulfur) on the photophysical properties. Since dyes 6a and 6b are ionic molecules, they are soluble in polar solvent like dimethyl sulfoxide (DMSO). Fig. 1 revealed the concentration-dependent UV-vis absorption spectra of 6a and 6b in DMSO. It was found that the absorption peak of 6a was 470 nm at 5 μM. In comparison, the dye 6b exhibited a peak at 475 nm, with a shoulder at approximately 580 nm, which is similar to that of unsymmetrical trimethine cyanine dyes reported by Ge and Lu *et al.*^44^ The absorption bands were slight bathochromic shift to longer wavelengths by increasing the concentration of 6a and 6b, which is attributed to the ground state aggregate species were formed in both 6a and 6b. The corresponding fluorescence emission spectra of 6a were obtained with excitation wavelength at 470 nm and displayed in Fig. 2a. As the concentration increased from 5 to 1000 μM, the emission intensities were enhanced *ca.* 1000 fold, indicating that the dye of 6a was AIE material. In contrast, the dual-wavelength emission was found to enhance emission intensities by increasing the concentration of 6b (Fig. 2b). The insets of Fig. 2 showed the photographs of solid powders of 6a and 6b taken under UV irradiation at 365 nm, with bright orange-red and red emissions, respectively. Additionally, the excitation spectra were recorded for the emission wavelength of 620 nm, and the higher the concentration, the higher the excitation spectrum intensity at the 580 nm wavelength (Fig. S1†).

**Fig. 3:**
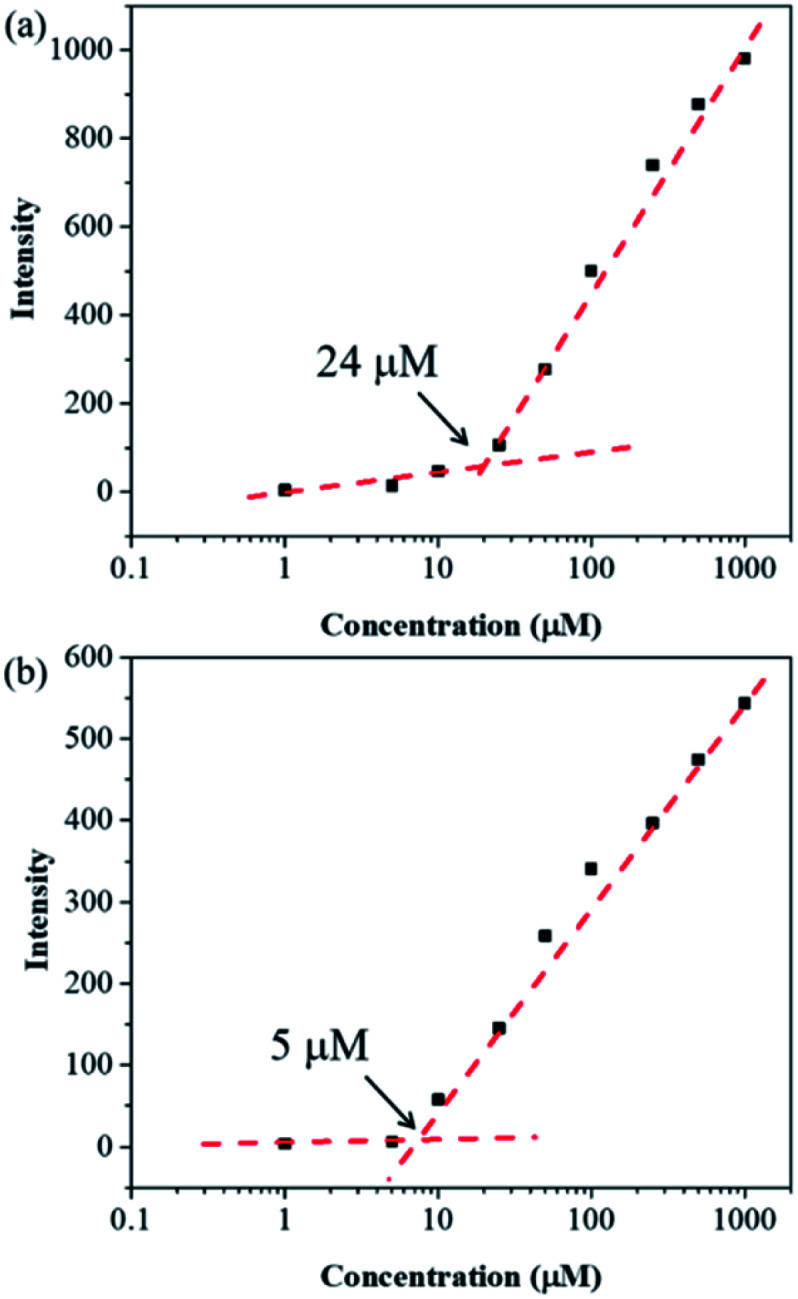
CAC of (a) 6a and (b) 6b in DMSO.

**Fig. 4:**
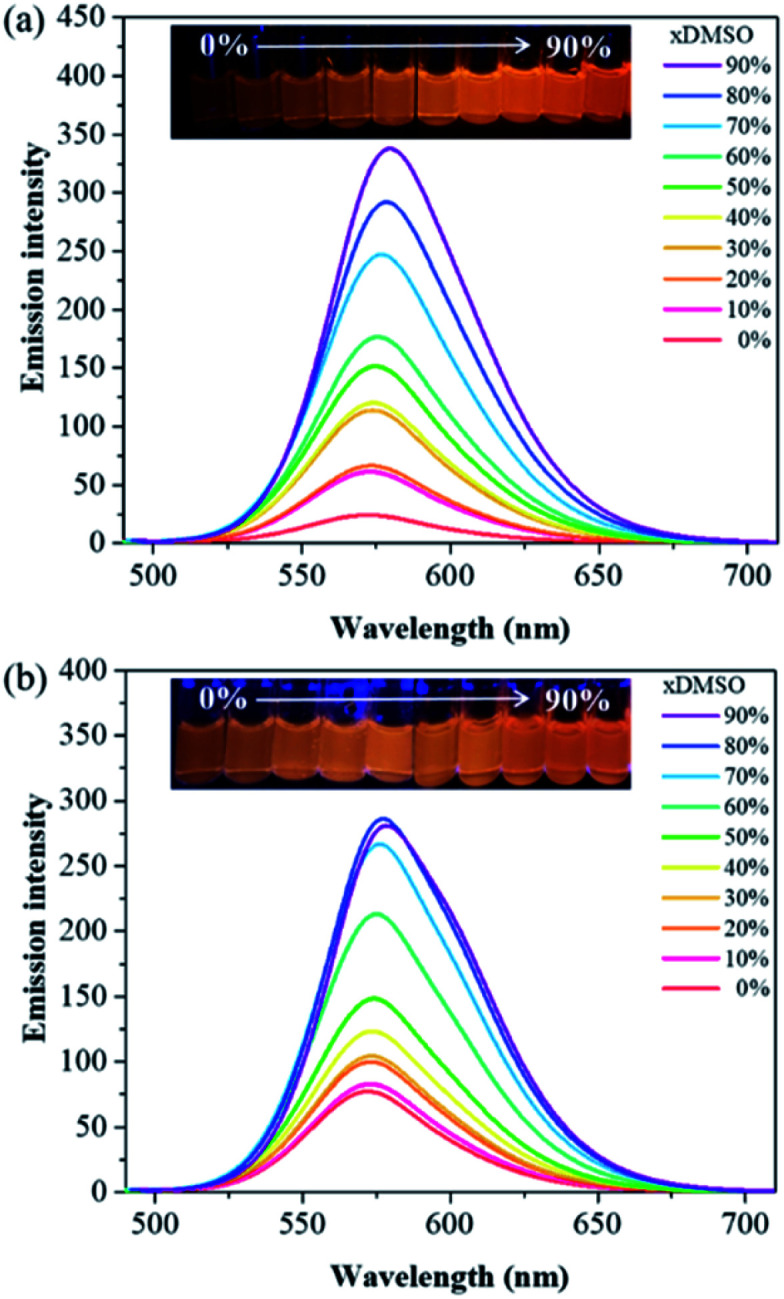
Fluorescence emission spectra of (a) 6a and (b) 6b in water/DMSO mixtures (concentration = 50 μM; *λ*_ex_ = 470 nm for 6a and *λ*_ex_ = 475 nm for 6b). The insets are the photographs of 6a and 6b in solvent mixtures of various fractions (*x*DMSO), taken under UV irradiation at 365 nm.

In order to get more deep understanding of the aggregate abilities and properties of dyes 6a and 6b, we studied their critical aggregation concentration (CAC) and the volume fraction of DMSO (*x*DMSO) in the binary water/DMSO mixtures. Fig. 3 presented that the CAC of 6a and 6b were 24 μM and 8 μM, respectively, suggesting that compound 6b has more tendency to aggregate than 6a in pure DMSO. The solubility of the molecule in a polar solvent can be confirmed from the dipole moment values. Therefore, the dipole moments of 6a and 6b cations were determined by two different calculation methods. The calculated dipole moments of 6a and 6b cations were 12.5897 D and 12.1788 D, respectively, by using DFT/B3LYP/6-31G(d,p) basis set. While the dipole moments calculated through HF/6-31G(d,p) method were found to be 8.8510 D for 6a cation and 8.2871 D for 6b cation. Moreover, the octanol–water partition coefficient (clog *P*) was also used to evaluate the polarity of compounds, and it is known that the more polar, hydrophilic compound will have a lower *c*log *P*.^45,46^ The *c*log *P* values of 6a and 6b cations were 1.2339 and 1.8299, respectively. These results suggested that compound 6a would be more polar than 6b, consequently leading to higher solubility in DMSO. To further examine the relationship between the ionic molecules and the solvent polarity, the various water/DMSO mixtures were conducted at 50 μM, based on the CAC evaluation of 6a and 6b in DMSO. As can be seen from Fig. 4, the emission intensities were increased in lower polarity systems, showing that the AIE characteristics in the aggregate states of 6a and 6b. It is worth noting that 6a with weak fluorescence intensity in pure water, owing to its polar molecule structure. By increasing the DMSO fraction, we can observe the considerable enhancement of the emission intensity (Fig. 4a). On the other hand, 6b exhibited less polarity with respect to 6a, which is expected to produce an aggregate state in water and result in bright emission (Fig. 4b). The calculation results of the dipole moments are comparable with the experimental observations.

### 3.3. Theoretical calculations

To achieve more insight into the intrinsic properties of the molecules 6a and 6b, molecular calculations were performed using the Gaussian program. The geometries were optimized at DFT/B3LYP/6-31G(d,p) level of theory. The optimized structures of 6a and 6b cations were displayed in Fig. S2,† the corresponding bond lengths, bond angles as well as dihedral angles were revealed in Table 1. The calculated bond lengths of C_10_–O and C_10_–S were 1.35 and 1.75 Å, respectively. The bond angles of C_10_–O–C_12_ and C_10_–S–C_12_ were 108.25° and 91.61°, respectively. These results suggest that the heteroatoms of oxygen and sulfur show a significant influence on the molecular structures of 6a and 6b. Furthermore, the dihedral angles of C_4_–C_5_–N_2_–C_7_, C_7_–C_8_–C_9_–C_10_ and C_10_–O–C_12_–C_13_ were in the range of 178.90° to 179.85° for 6a; C_4_–C_5_–N_2_–C_7_, C_7_–C_8_–C_9_–C_10_ and C_10_–S–C_12_–C_13_ were in the range of 179.21° to 179.92° for 6b. These values of dihedral angles for 6a and 6b indicated that they are π-conjugated compounds. Notably, the dihedral angles of the phenyl rings at 4, 5-position of imidazole and imidazole core were in the range of 28.85° to 55.69° for 6a and 6b (Fig. S2†), implying that the twisted conformations would be responsible for the AIE characteristic of dyes 6a and 6b (Fig. 2 and 4).^23^ Meanwhile, HF/6-31G(d,p) method was achieved for comparison with DFT results, the calculated bond lengths, bond angles and dihedral angles values obtained with DFT and HF are comparable (Fig. S2† and Table 1).

**Table 1:** Selected bond lengths (R, Å), bond angles (D, °) and dihedral angles (Di, °) of 6a^+^ and 6b^+^^a^

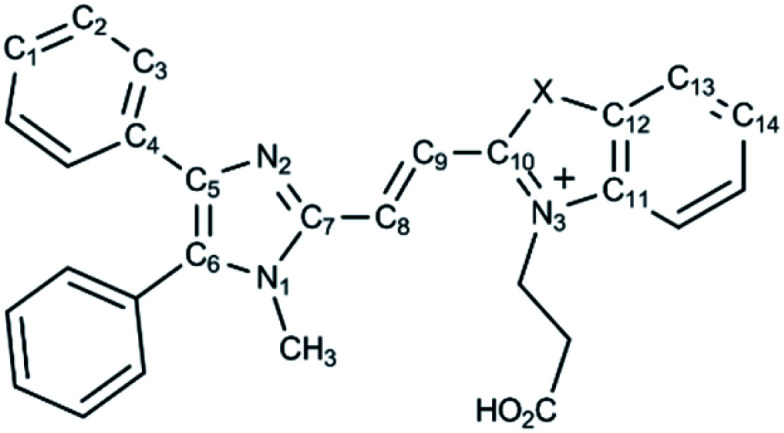				
	6a^+^ (DFT)^b^	6b^+^ (DFT)^b^	6a^+^ (HF)^c^	6b^+^ (HF)^c^
R (C_1_–C_2_)	1.397	1.397	1.386	1.386
R (C_2_–C_3_)	1.392	1.392	1.383	1.383
R (C_3_–C_4_)	1.406	1.406	1.392	1.392
R (C_4_–C_5_)	1.472	1.471	1.480	1.480
R (C_5_–C_6_)	1.428	1.427	1.391	1.390
R (C_6_–N_1_)	1.364	1.365	1.355	1.357
R (N_1_–C_7_)	1.392	1.391	1.362	1.361
R (C_5_–N_2_)	1.344	1.345	1.339	1.341
R (N_2_–C_7_)	1.342	1.342	1.308	1.308
R (C_7_–C_8_)	1.414	1.416	1.433	1.435
R (C_8_–C_9_)	1.381	1.383	1.351	1.351
R (C_9_–C_10_)	1.404	1.412	1.426	1.434
R (C_10_–N_3_)	1.359	1.362	1.324	1.324
R (N_3_–C_11_)	1.405	1.407	1.407	1.410
R (C_11_–C_12_)	1.392	1.404	1.371	1.383
R (C_10_–X)	1.352	1.753	1.312	1.726
R (X–C_12_)	1.380	1.753	1.368	1.744
R (C_12_–C_13_)	1.383	1.395	1.374	1.387
R (C_13_–C_14_)	1.398	1.392	1.386	1.378
D (C_5_–N_2_–C_7_)	107.11	107.14	106.98	106.95
D (C_6_–N_1_–N_7_)	107.25	107.28	106.65	106.66
D (C_10_–N_3_–C_11_)	108.49	114.77	107.91	114.33
D (C_10_–X–C_12_)	108.25	91.61	108.77	91.31
Di (C_4_–C_5_–N_2_–C_7_)	179.22	179.21	179.99	179.97
Di (C_7_–C_8_–C_9_–C_10_)	178.90	179.52	179.90	178.53
Di (C_10_–X–C_12_–C_13_)	179.85	179.92	179.91	179.89

a For 6a^+^, X is oxygen atom; for 6b^+^, X is sulfur atom.

b DFT/B3LYP/6-31G(d,p).

c HF/6-31G(d,p).

**Fig. 5:**
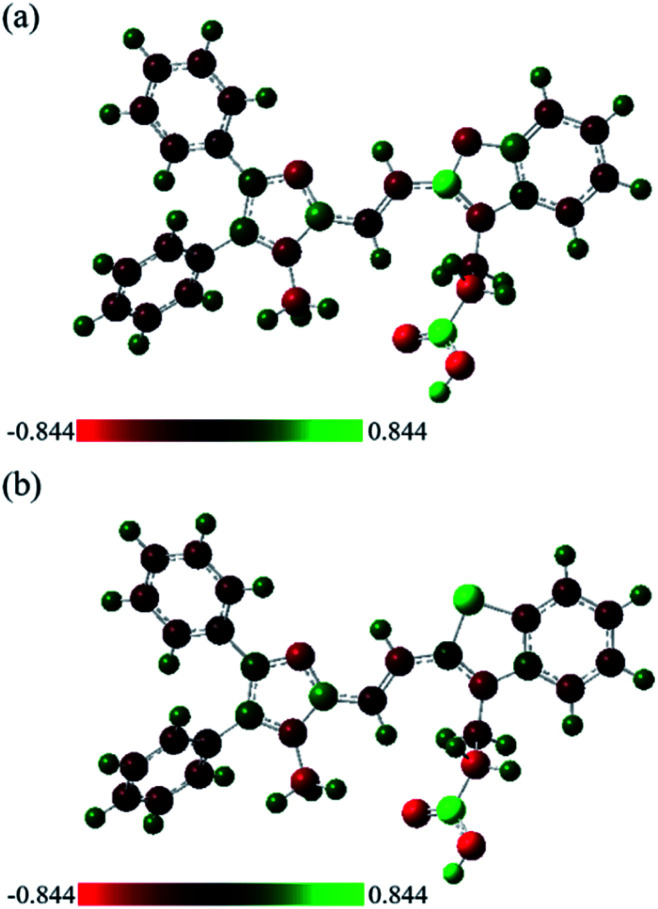
NBO charge distribution on the optimized (a) 6a^+^ and (b) 6b^+^ designated by color change on the atoms with color scheme and scale (red for negative charge and green for positive charge), determined using DFT at the B3LYP/6-31G(d,p) level.

Since the oxygen and sulfur substitutions can dramatically influence molecular structures of 6a and 6b, the natural bond orbital (NBO) analysis was used to further investigate the charge delocalization and noncovalent interactions.^47^ NBO study offers an efficient method for investigating intra- and intermolecular bonding and interaction among bonds. It provides a convenient basis for understanding the arrangement of molecules in the aggregate states^.^^48,49^ As can be seen from Table 2, the NBO charge of the oxygen atom was −0.43, while the charge of the sulfur atom was 0.54 using DFT calculation (the HF method had the same trend as the DFT data). Moreover, the NBO charges of the three nitrogen atoms of 6a and 6b were in the range from −0.33 to −0.45, suggesting that it might have electrostatic interaction between sulfur and nitrogen atoms. It is probably due to the opposite charges of the heteroatoms in 6b, leading to an increase the tendency to aggregate and the formation of the dual emission. The visualization of charge distributions of 6a and 6b were depicted in Fig. 5 and S3,† it was clear to perceive that the positive and negative charged electrostatic potential in the molecules. The NBO charge analysis presented here explains the experimental observations of CAC and photophysical properties both in solution and in the solid state (Fig. 2–4).

**Table 2:** Selected NBO charge distribution of 6a^+^ and 6b^+^^a^

	6a^+^ (DFT)^b^	6b^+^ (DFT)^b^	6a^+^ (HF)^c^	6b^+^ (HF)^c^
C_1_	−0.21663	−0.21670	−0.21268	−0.21305
C_2_	−0.23122	−0.23140	−0.23099	−0.23111
C_3_	−0.19996	−0.20031	−0.19494	−0.19557
C_4_	−0.09745	−0.09674	−0.10388	−0.10296
C_5_	0.17663	0.17671	0.16744	0.16804
C_6_	0.22322	0.22190	0.26219	0.25901
C_7_	0.32061	0.32588	0.30455	0.31226
C_8_	−0.17008	−0.17616	−0.04400	−0.05185
C_9_	−0.35870	−0.33164	−0.45544	−0.42530
C_10_	0.61046	0.08347	0.79425	0.21800
C_11_	0.11319	0.14996	0.12736	0.17738
C_12_	0.29262	−0.22928	0.31879	−0.24872
C_13_	−0.25362	−0.22158	−0.24457	−0.20471
C_14_	−0.21563	−0.21512	−0.21352	−0.21879
N_1_	−0.33428	−0.33496	−0.40310	−0.40407
N_2_	−0.44915	−0.45462	−0.46350	−0.47120
N_3_	−0.37692	−0.35749	−0.47700	−0.45253
X	−0.42640	0.53972	−0.52358	0.52132

a For 6a^+^, X is oxygen atom; for 6b^+^, X is sulfur atom.

b DFT/B3LYP/6-31G(d,p).

c HF/6-31G(d,p).

**Fig. 6:**
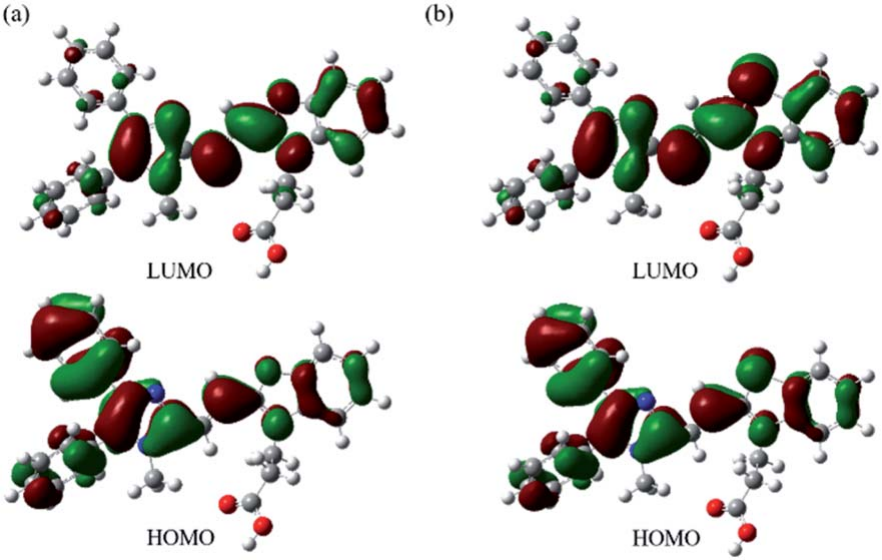
Graphical demonstration of HOMO and LUMO energy levels of (a) 6a and (b) 6b calculated by using DFT at the B3LYP/6-31G(d,p) level.

Furthermore, from the calculated results of the dihedral angles in Table 1, we note that the compounds of 6a and 6b could be π-conjugated molecules. Therefore, we observed the electron densities of their highest occupied molecular orbitals (HOMO) are distributed in the whole molecule backbone, and that of their lowest unoccupied molecular orbitals (LUMO) are located on the core of imidazole and benzoxazolyl unit (as well as the core of imidazole and benzothiazolyl unit). Compared with an oxygen atom, the sulfur atom has a noticeable contribution to its HOMO and LUMO components (Fig. 6). Table S1† summarizes the TD-DFT results for the lowest energy vertical excitation. The transition energies of 6a and 6b cations obtained with DFT/B3LYP/6-31G(d,p) were 2.3319 eV and 2.2435 eV, respectively, indicating that compound 6b exhibits longer wavelength absorption than that of 6b. Additionally, HF/6-31G(d,p) was also tested for 6a and 6b, which provides qualitatively similar results. The calculation results obtained by DFT/B3LYP/6-31G(d,p) and HF/6-31G(d,p) agree with the experimental data of their optical properties.

## 4. Conclusions

In summary, we have newly synthesized the benzoxazolyl-imidazole and benzothiazolyl-imidazole conjugates, which show aggregation-induced emission (AIE) features in their solid and aggregate states. The effect of heteroatoms (oxygen and sulfur) on the photophysical properties as well as molecular geometries were investigated. It was found that the oxygen and sulfur substitutions can dramatically influence molecular structures and polarities of the dyes, leading to the different degree of AIE phenomenon. Since the benzothiazolyl-imidazole molecule has lower polarity than benzoxazolyl-imidazole, the dye bearing benzothiazolyl group shows higher emission intensity and dual emission in an aqueous solution, which accompanying the more minor AIE enhancement and red solid is observed. Theoretical calculation results suggest that the benzothiazolyl-imidazole molecule might have electrostatic interaction between sulfur and nitrogen atoms, explaining the experimental observations of lower critical aggregation concentration and photophysical properties both in solution and solid state. The theoretical calculations are in agreement with the experimental data, thus demonstrating a potent strategy to gain a deep understanding of the structure–property relationship to design solid-state fluorescent materials.

## Conflicts of interest

There are no conflicts to declare.

## Supplementary Material

RA-011-D1RA08342B-s001
