# Supplementary material for: Synthesis and photophysical properties of benzoxazolyl-imidazole and benzothiazolyl-imidazole conjugates
Source: RSC Adv. 2021 Dec 17;11(63):40228–34. doi: 10.1039/d1ra08342b (PMC9044771; doi:10.1039/d1ra08342b)
Supplement: RA-011-D1RA08342B-s001 [file RA-011-D1RA08342B-s001.pdf]

Electronic Supplementary Material (ESI) for RSC Advances.
This journal is © The Royal Society of Chemistry 2021

Synthesis and photophysical properties of benzoxazolyl-imidazole and benzothiazolyl-imidazole conjugates

Hsing-Yu Chen, Chen-Chen Yao, Tzu-Yu Tseng, Yao-Chun Yeh, He-Shin Huang and Mei-Yu Yeh*

Department of Chemistry, Chung Yuan Christian University, No. 200, Zhongbei Rd., Zhongli Dist., Taoyuan City 320314, Taiwan

Contents **Page Number**

1. Fig S1. Excitation spectra 2
2. Fig S2. The geometrical optimization structures 2
3. Fig S3. NBO charge distribution 3
4. Table S1 3
5. Fig. S4 and S5 ^{1}H NMR spectra 4
6. Fig. S6 and S7 HRMS spectra 5

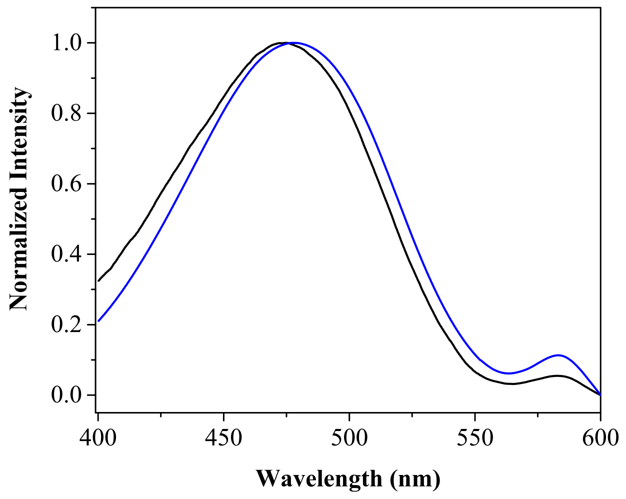

Fig. S1 Excitation spectra of **6b** with excitation wavelength at 620 nm. (black: 100 μM; blue: 500 μM)

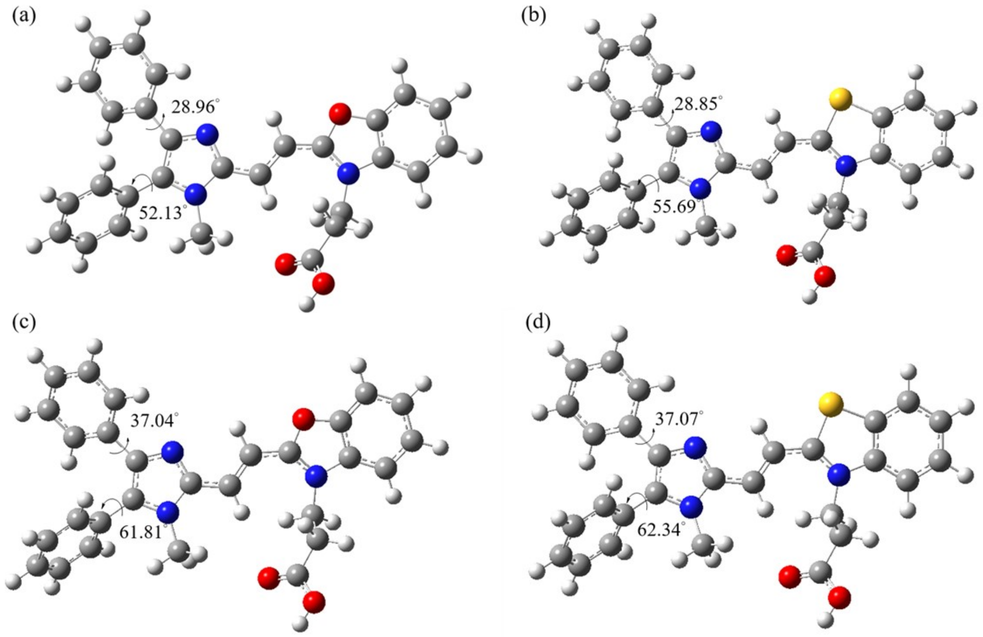

Fig. S2 The geometrical optimization structures of (a, c) **6a**$^{+}$ and (b, d) **6b**$^{+}$. (a, b) calculated by using DFT/B3LYP/6-31G (d,p); (c, d) calculated by using HF/6-31G (d,p). C: gray; H: white; N: blue; O: red; S: yellow.

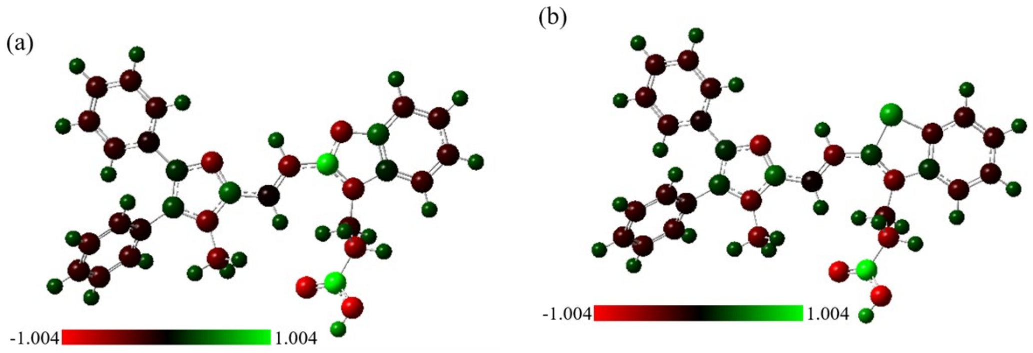

Fig. S3. NBO charge distribution on the optimized (a) **6a**$^{+}$ and (b) **6b**$^{+}$ designated by color change on the atoms with color scheme and scale (red for negative charge and green for positive charge), determined using HF/6-31G (d,p) level.

Table S1 Absorption spectra were obtained using TD-DFT methods for **6a**$^{+}$ and **6b**$^{+}$.[a]

	Optimized Geometries	Absorption (eV)	Electronic transitions	Oscillator strength
**6a**$^{+}$	DFT[b]	2.3319	H→L (0.69155)	0.7662
	HF[c]	2.3954	H→L (0.62171)	0.5869
**6b**$^{+}$	DFT[b]	2.2435	H→L (0.69333)	0.8094
	HF[c]	2.2707	H→L (0.61790)	0.6040

[a] For **6a**$^{+}$, X is oxygen atom; for **6b**$^{+}$, X is sulfur atom; [b] DFT/B3LYP/6-31G (d,p); [c] HF/6-31G (d,p).

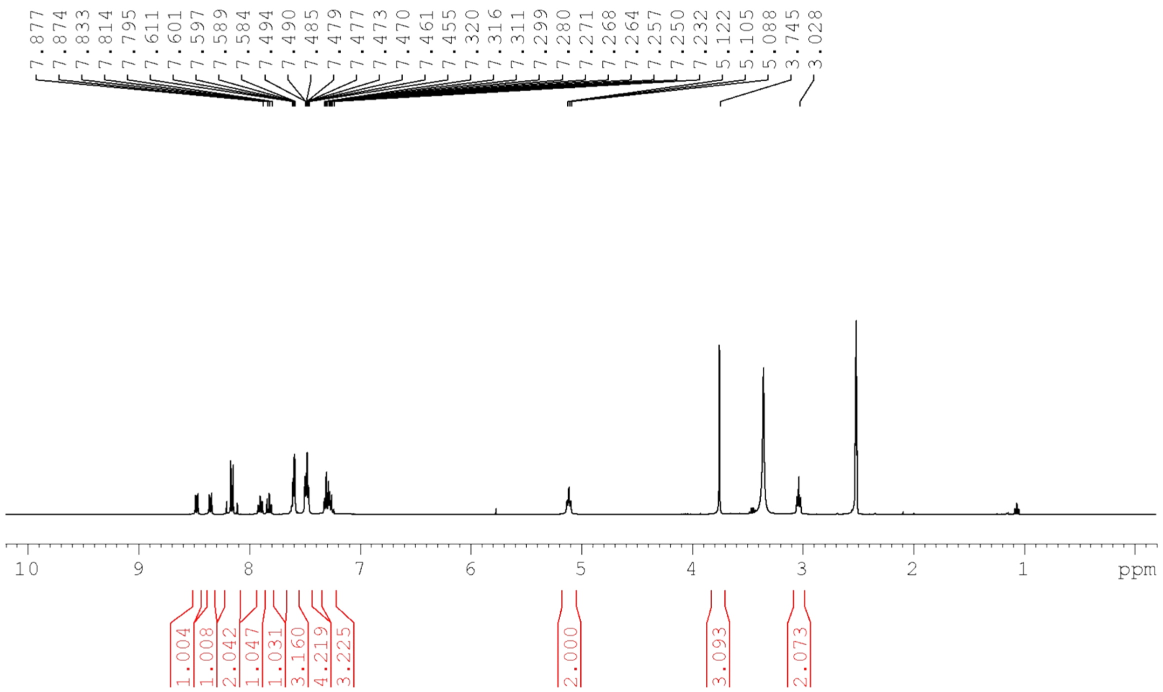

Fig. S4 ^{1}H NMR spectrum for **6a** in DMSO-d_6.

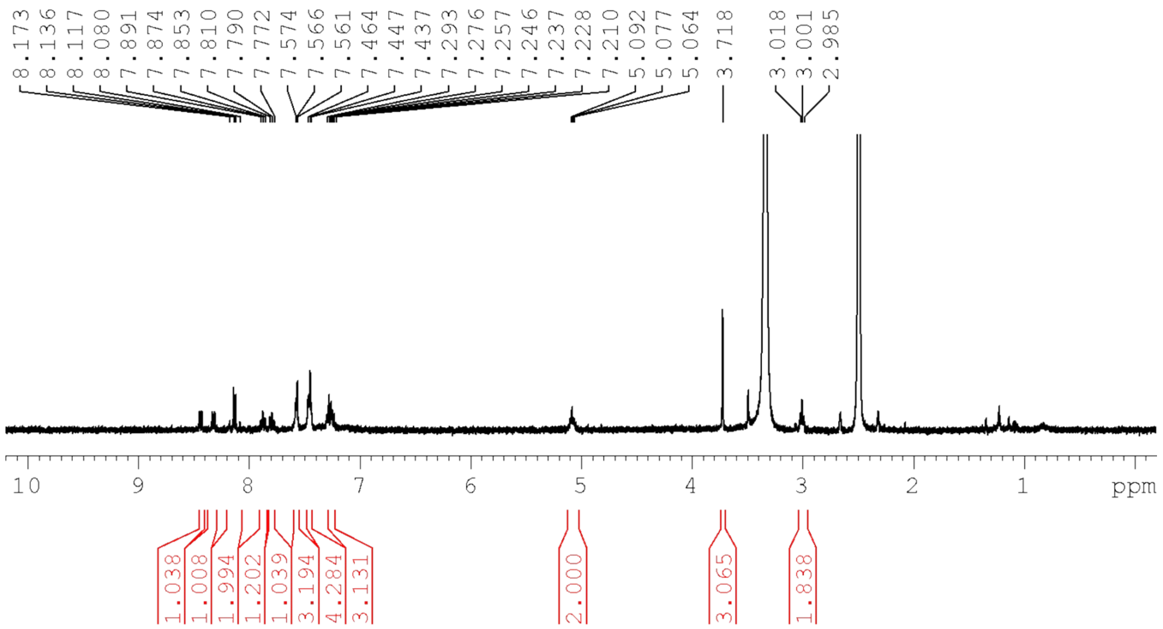

Fig. S5 ^{1}H NMR spectrum for **6b** in DMSO-d_6.

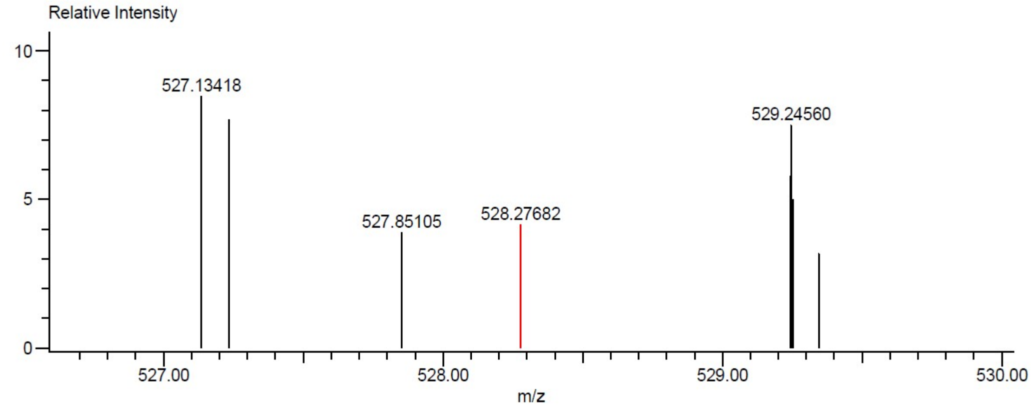

Mass	Intensity	Calc. Mass	Mass Difference [mDa]	Mass Difference [ppm]	Possible Formula
528.27682	82.57	528.09228	184.55	349.46	$^{12}C_{28}{}^{1}H_{23}{}^{79}Br_{1}{}^{14}N_{3}{}^{16}O_{3}$

Fig. S6. HRMS spectrum for **6a**.

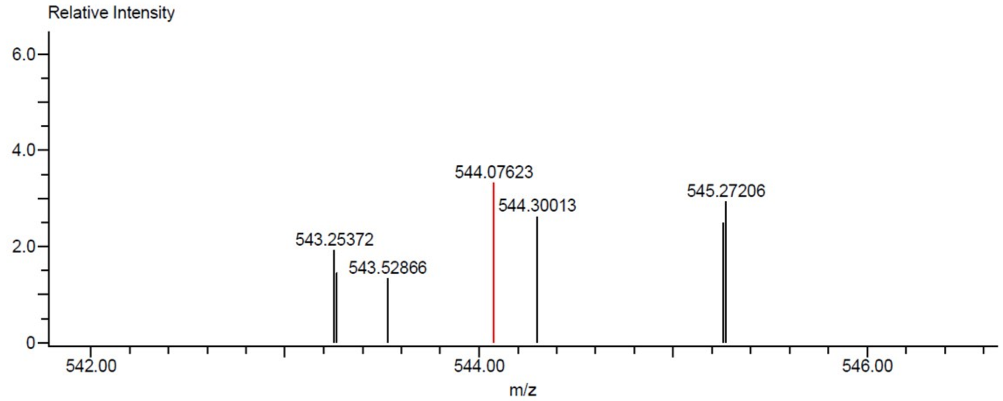

Mass	Intensity	Calc. Mass	Mass Difference [mDa]	Mass Difference [ppm]	Possible Formula
544.07623	201.52	544.06943	6.79	12.49	$^{12}C_{28}{}^{1}H_{23}{}^{79}Br_{1}{}^{14}N_{3}{}^{16}O_{2}{}^{32}S_{1}$

Fig. S7. HRMS spectrum for **6b**.
